# Child Nutrition in the Aspirational Districts of Uttar Pradesh, India: Findings From the National Family Health Survey-5, 2019-21

**DOI:** 10.7759/cureus.102521

**Published:** 2026-01-28

**Authors:** Ajay Pandey, Richa Sharma

**Affiliations:** 1 Population Research Center, University of Lucknow, Lucknow, IND; 2 Research, National Institute of Labour Economics Research and Development, New Delhi, IND

**Keywords:** aspirational districts, child malnutrition, low weight-for-height, nfhs-5, niti aayog, stunting, underweight, uttar pradesh, wasting

## Abstract

The Aspirational Districts Programme (ADP) of the Government of India aims to quickly and effectively transform 112 of the most underdeveloped districts across the country through scheme convergence, collaboration, and competition among districts. Nutrition being a key component and Uttar Pradesh being the largest state in the country, this study aims to analyze the child nutrition levels in eight aspirational districts of the state using the National Family Health Survey (NFHS-5), 2019-21, data. Finding suggests stunting rates in the Aspirational Districts (45.08%) are substantially higher than the average for all districts of Uttar Pradesh (39.71%). Similarly, children in the Aspirational Districts of the state experience higher wasting rates (20.35%) compared to the state average of 17.32%. Across all Aspirational Districts, 38.07% of children are underweight, compared to 32.14% for the state overall. Similarly, 15.65% of children in Aspirational Districts are severely underweight, compared to just 11.13% across the state. A multivariate analysis of the effects of selected demographic and socioeconomic factors on child malnutrition indicates that the strongest predictors of child nutrition in India are the child’s age and birth order, mother’s education, and household socioeconomic status in terms of wealth quintiles. Older children and children of higher birth order are more likely to be malnourished. Children whose mothers are more educated and children who live in households with relatively high wealth quintiles tend to be better nourished than other children.

## Introduction

Malnutrition, encompassing stunting (low height-for-age), wasting (low weight-for-height), and underweight (low weight-for-age), reflects the cumulative effects of inadequate nutrition, poor health services, and socio-economic marginalization [1]. Child malnutrition remains one of the most pressing public health challenges in India [2], particularly in Aspirational Districts, which are identified by the NITI (National Institution for Transforming India) Aayog as the most socially and economically underdeveloped regions in the country [3].

Uttar Pradesh, India's most populous state, consistently reports some of the highest levels of child malnutrition in the country, with National Family Health Survey (NFHS-5), 2019-21, data indicating that 39.7% of children under five are stunted, 19.5% wasted, and 32.1% underweight [2]. These rates far exceed national averages and highlight the severe nutritional challenges faced by children in the state, particularly in Aspirational Districts, which lag behind on development indicators, including health, nutrition, education, and basic infrastructure.

The importance of tackling malnutrition in the early years of life cannot be overstated. Childhood malnutrition has long-term effects on physical and cognitive development, school performance, and economic productivity in adulthood [4]. The first 1,000 days, from conception to a child’s second birthday, represent a critical window for ensuring optimal growth and development. Failure to provide adequate nutrition and care during this period results in irreversible damage to a child’s physical and cognitive potential [5].

The nutritional status of children is a direct reflection of household and maternal factors, including maternal health, education, and access to antenatal care, as well as community-level determinants such as poverty, caste-based discrimination, access to safe water and sanitation, and food security [6]. Despite India’s substantial progress in reducing poverty and improving access to health services, large pockets of undernutrition persist, particularly among Scheduled Castes (SC), Scheduled Tribes (ST), and Other Backward Classes (OBC) [7]. These groups face structural disadvantages that limit their access to resources, services, and economic opportunities, perpetuating intergenerational cycles of malnutrition and poverty [8].

Children who are stunted by age two are more likely to have poor cognitive and educational outcomes, lower economic productivity in adulthood, and increased risk of non-communicable diseases (NCDs) later in life [1]. The persistence of high stunting rates in Uttar Pradesh’s Aspirational Districts reflects the cumulative burden of food insecurity, maternal undernutrition, inadequate infant feeding practices, and poor access to health services [2].

Wasting, characterized by low weight-for-height, is often triggered by acute episodes of food insecurity, disease outbreaks (especially diarrhea and respiratory infections), or shocks such as floods or droughts [9]. The high prevalence of wasting in Uttar Pradesh (19.5%) exceeds the 15% emergency threshold set by the World Health Organization [10], underscoring the severity of acute malnutrition in the state. Unlike stunting, wasting can develop quickly in response to short-term crises, making it a sensitive indicator of recent dietary and health shocks [11].

The third indicator, underweight, captures both chronic and acute undernutrition. Its strong association with poverty, maternal illiteracy, and household food insecurity has been extensively documented [6,8]. The NFHS-5 highlighted that children in Uttar Pradesh’s poorest households were twice as likely to be underweight compared to those in the wealthiest quintile [2]. This wealth gradient is consistent with global evidence, which shows that malnutrition is both a cause and consequence of poverty [1].

Numerous studies emphasize the critical role of maternal education in improving child nutrition outcomes [12]. Educated mothers are more likely to adopt appropriate infant feeding practices, ensure timely immunization, and seek healthcare when needed [13]. In Uttar Pradesh, the NFHS-5 data show a clear inverse relationship between maternal education and stunting, wasting, and underweight, a finding echoed in studies across South Asia [2,6].

Maternal health and nutrition during pregnancy also play a central role in determining birth weight and early growth trajectories [5]. Iron and folic acid (IFA) supplementation and tetanus immunization during pregnancy contribute to better birth outcomes, but their direct link to long-term nutritional status is less pronounced [11]. The broader household environment, including household wealth, food security, and access to safe water and sanitation, further influences child growth [9].

The persistent caste-based disparities in malnutrition observed in Uttar Pradesh are consistent with findings across India, where children from SC and ST communities face systematically higher rates of undernutrition [7]. These inequalities are rooted in historical marginalization, restricted access to productive assets, poor living conditions, and discriminatory access to health and nutrition services [14]. Addressing these structural inequities is essential to improving nutrition outcomes in marginalized communities.

Although undernutrition remains the dominant challenge, emerging evidence points to a growing double burden where overweight and obesity coexist with undernutrition, particularly in urban areas and wealthier households [15]. Changes in dietary patterns, increased consumption of processed foods, and reduced physical activity are contributing to rising overweight rates even in rural areas [16]. This nutrition transition presents a new challenge for policymakers, who must simultaneously address severe undernutrition and emerging overnutrition risks.

This study focuses on the nutritional status of children aged 0-59 months in the Aspirational Districts of Uttar Pradesh, analyzing the prevalence and determinants of stunting, wasting, and underweight. By identifying the socio-economic, demographic, and health service factors associated with these forms of malnutrition, this study aims to inform evidence-based interventions to improve child nutrition outcomes in Uttar Pradesh’s most vulnerable districts.

## Materials and methods

This study is based on the NFHS-5 (2019-21) data [2]. The analysis was carried out at the Population Research Centre at the University of Lucknow, Uttar Pradesh, India. The Population Research Centre is a Ministry of Health and Family Welfare, Government of India-funded research centre.

Study population

The NFHS-5 provides information on population, health, and nutrition for 707 districts in India. Among the data files, the Persons Record File (also known as Household Members Recode) is a dataset where each row represents an individual household member. In the NFHS, the nutrition status of the children was analyzed using information collected on the height and weight of children in different age groups using the Persons Record File. 

Data for undernourished and malnourished children, aged 0-5 years, from the eight Aspirational Districts of Uttar Pradesh (Bahraich, Balrampur, Banda, Chandauli, Chitrakoot, Fatehpur, Shrawasti, Siddharthnagar) [3] were used in the estimation of the child nutrition indices. The total unweighted sample size was 3885. In this study, we used the weighted sample, resulting in a total of 3881.

Child nutrition indices

Each of these indices studied provides somewhat different information about the nutritional status of children. The height-for-age index measures linear growth retardation among children, primarily reflecting chronic malnutrition. The weight-for-height index measures body mass in relation to body height, primarily reflecting acute malnutrition. Weight-for-age reflects both chronic and acute malnutrition. The calculation of the three indices of child malnutrition involves comparison with an international reference population as recommended by the World Health Organization [17,18]. The definitions as per the NFHS-5 are given below.

Stunting (Assessed via Height-for-Age)

Height-for-age is a measure of linear growth retardation and cumulative growth deficits. Children whose height-for-age Z-score < -2 SD from the median of the reference population are considered short for their age (stunted), or chronically undernourished. Children who are < -3 SD are considered severely stunted [2].

Wasting (Assessed via Weight-for-Height)

The weight-for-height index measures body mass in relation to body height or length and describes current nutritional status. Children whose Z-score is < -2 SD from the median of the reference population are considered thin (wasted), or acutely undernourished. Children whose weight-for-height Z-score is < -3 SD from the median of the reference population are considered severely wasted [2].

Underweight (Assessed via Weight-for-Age)

Weight-for-age is a composite index of height-for-age and weight-for-height. It takes into account both acute and chronic undernutrition. Children whose weight-for-age Z-score is < -2 SD from the median of the reference population are classified as underweight. Children whose weight-for-age Z-score is < -3 SD from the median are considered severely underweight [2].

Overweight

Children whose weight-for-height Z-score is > 2 SD above the median of the reference population are considered overweight [2].

Data analysis

As many of the factors associated with child nutrition are also associated with each other, to address the issue of confounding, multivariate analysis was carried out to study the effects of demographic and socioeconomic variables on the three indices of child malnutrition using logistic regression models. The response (or dependent) variable in each model was a dummy (two-category) variable that simply indicated whether a child was stunted, underweight, or wasted. The predictor variables were: child’s age in months, sex of child, birth order of child, mother’s age at childbirth, residence (urban, rural), mother’s education (illiterate, literate but less than middle school complete, middle school complete or higher), religion (Hindu, Muslim, other), caste/tribe (SC, ST, OBC), exposure to electronic mass media (regularly exposed, not regularly exposed), wealth quintile, mother received IFA tablets during pregnancy, and mother received two or more tetanus toxoid (TT) injections during pregnancy. Stata Statistical Software: Release 16 (StataCorp LLC., College Station, Texas, United States) was used for the analysis.

## Results

Anthropometric measures of a weighted sample of 3881 children aged 0-5 years from the eight Aspirational districts of Uttar Pradesh were included in the analysis to determine the prevalence and determinants of stunting, wasting, underweight, and overweight.

Stunting

Table 1 presents data on the percentage of children aged 0-59 months who are classified as severely stunted or stunted across the Aspirational Districts of Uttar Pradesh for the period 2019-21.

**Table 1:** Demographic characteristics of children age 0–59 months, the percentage classified as undernourished according to three anthropometric indices of nutritional status ^a^ below the international reference population median; * fewer cases SC: scheduled castes; ST: scheduled tribes; OBC: other backward classes; TT: tetanus toxoid; UP: Uttar Pradesh

Background Variables	Severely Stunted	Stunted	Severely wasted	Wasted	Severely Underweight	Underweight	Overweight for height	Overweight for age	Overall Sample
	Percentage < -3 SD	Percentage < -2 SD^a^	Percentage < -3 SD	Percentage < -2 SD^a^	Percentage < -3 SD	Percentage < -2 SD^a^	Percentage > 2 SD	Percentage > 2 SD^a^	N
District									
Bahraich	29.59	52.11	7.47	14.35	14.49	38.04	4.13	1.26	938
Balrampur	19.26	41.15	14.27	24.93	19.96	37.16	3.9	1.92	524
Chitrakoot	25.24	47.45	12.02	24.82	19.61	41.78	6.72	1.54	161
Chandauli	14.28	39.48	7.44	17.43	11.63	29.94	0.57*	0.00*	413
Fatehpur	27.85	51.06	9.06	17.82	15.09	37.97	3.63	0.99*	481
Shrawasti	30.01	50.93	9.31	20.31	18.24	40.76	3.33	0.40*	344
Sidharthnagar	17.24	37.17	14.47	24.84	14.28	36.32	2.79	0.74*	611
Sonbhadra	15.65	38.26	17.36	26.75	18.29	46.49	0.87	0.23*	409
Child's Age (in months)									
<12	12.37	24.09	14.37	28.05	13.53	28.11	5.98	2.46	751
12-23	23.89	43.82	11.05	23.05	14.77	35.66	3.82	0.92	780
24-59	25.74	52.16	9.91	17.07	16.74	42.32	2.11	0.43	2350
Sex of the Child									
Male	23.41	45.27	11.28	21.31	16.79	38.95	3.12	0.93	2016
Female	21.94	44.55	10.67	19.44	14.43	37.12	3.24	0.96	1865
Birth Order									
One	19.4	41.3	11.29	19.71	14.66	36.64	3.15	1.11	1099
Two, three	21.84	43.91	11.39	21.51	15.74	37.51	2.6	0.96	1800
Four, Five	27.79	51.58	9.82	18.7	16.59	41.12	4.58	0.67	733
Six or more	29.5	50.71	10.25	20.13	17.22	40	3.4	0.85	249
Mother's age at child’s birth									
13-24	21.18	44.41	11.56	20.38	16.73	38.84	2.68	0.85	1832
25-34	23.43	45.24	10.25	19.8	14.49	36.97	3.39	1.06	1766
35-59	29.14	48.16	11.9	23.84	16.52	40.53	5.27	0.82	283
Mother's Education									
Illiterate	28.01	50.81	11.4	21.02	18.29	42.92	3.61	1.31	1792
Primary	22.37	47.51	10.98	19.99	15.6	36.55	2.33	0.35	561
Secondary	18.34	39.98	11.16	20.88	13.12	35.13	2.36	0.53	1163
Higher	11.85	29.2	8.43	16.08	11.28	26.34	5.16	1.35	365
Social Category									
SC/ST	26.51	49.77	11.76	21.32	16.66	43.4	2.68	0.97	1160
OBC	22.33	44.4	10.46	20.28	16.06	37.17	3.18	0.7	2069
General	18.12	38.83	11.5	18.87	12.72	31.59	4.02	1.48	645
Household Religion									
Hindu	22.08	44.7	10.91	20.44	15.76	38.18	2.81	0.86	2933
Muslim	25.4	46.65	11.37	20.17	15.43	37.93	4.13	1.16	939
Others	0	5.73	0	10.83	0	16.56	18.7	0	9
Place of Residence									
Urban	15.29	34.59	14.37	26.88	15.25	32.14	1.26	0.52	386
Rural	23.66	46.23	10.62	19.63	15.69	38.71	3.37	0.98	3495
Wealth quintile									
Lowest	27.94	51.68	11.2	21.29	17.82	43.65	3.39	0.88	1867
Second	21.99	45.08	11.94	20.06	15.4	37.89	3.27	1.14	1031
Middle	15.64	36.07	10.2	20.83	14.94	31.74	2.66	0.68	487
Fourth	13.84	29.94	7.57	16.17	8.59	26.14	2.48	0.67	308
Highest	9.41	26.92	11.49	18.28	8.38	18.5	2.68	1.4	188
IFA use									
No	26.62	47.01	12.33	19.31	16.96	38.01	3.68	1.34	461
Yes	21.45	40.92	11.39	22.31	14.64	35.4	4.01	1.25	2300
TT injection									
<2 shots	23.85	42.94	10.74	21.88	15.24	35.46	5.7	1.35	598
>=2 shots	21.88	41.66	11.78	21.79	14.96	35.93	3.47	1.24	2162
Aspirational Districts of UP	22.83	45.08	10.99	20.35	15.65	38.07	3.2	0.93	3881
UP, excluding Aspirational Districts	17.32	39.1	6.9	16.99	10.62	31.47	3.1	0.77	24694
										Total	17.88	39.71	7.31	17.32	11.13	32.14	3.1	0.79	38623

There is considerable variation in stunting prevalence across districts. Among the Aspirational Districts, Bahraich reports the highest prevalence of stunting at 52.11%, followed closely by Fatehpur (51.06%) and Shrawasti (50.93%). These three districts also have some of the highest rates of severe stunting, indicating a severe nutrition crisis in these areas. In contrast, Sidharthnagar (37.17%), Sonbhadra (38.26%), and Chandauli (39.48%) show lower levels of stunting, although these rates are still concerning. The high levels of stunting in districts like Bahraich and Shrawasti could be linked to persistent poverty, poor infrastructure, limited healthcare access, and food insecurity.

There is a clear and concerning trend where stunting increases with age. Among infants under 12 months, only 24.09% are stunted, but this figure rises sharply to 43.82% for children aged 12-23 months, and further to 52.16% for children aged 24-59 months. This progression suggests that early infancy may benefit from breastfeeding protections, but as children transition to complementary foods, inadequate diets and exposure to infections likely contribute to poor growth outcomes. This emphasizes the need for improved complementary feeding practices, better dietary diversity, and household food security.

There is a small gender difference in stunting rates, with male children having a slightly higher stunting rate (45.27%) compared to female children (44.55%). While the gap is narrow, it may indicate subtle gender-based differences in feeding practices, healthcare access, or parental attention, although further research would be needed to confirm these factors.

Stunting rates tend to increase with higher birth order. First-born children have a stunting rate of 41.3%, which rises to 43.91% for second and third children, 51.58% for fourth and fifth children, and 50.71% for children of birth order six or higher. This pattern is consistent with the well-documented phenomenon that parental resources (time, attention, finances) tend to get stretched thin in larger families, which often leads to poorer health and nutrition outcomes for later-born children.

Stunting rates also vary by the mother's age at childbirth. Children born to younger mothers (aged 13-24 years) have a stunting rate of 44.41%, while those born to mothers aged 25-34 years show a slightly higher rate of 45.24%. The highest stunting rate (48.16%) is observed among children born to mothers aged 35-59 years, which may reflect the cumulative impacts of maternal undernutrition, reproductive stress, and higher parity in older mothers.

There is a strong inverse relationship between maternal education and stunting. Among children of illiterate mothers, the stunting rate is 50.81%, which gradually decreases to 47.51% for mothers with primary education, 39.98% for those with secondary education, and 29.2% for mothers with higher education. This confirms that maternal education plays a critical role in improving child nutrition outcomes, likely by enhancing awareness of proper feeding, hygiene, and healthcare-seeking practices.

Social inequities in child nutrition are evident in the data. Stunting rates are highest among SC and ST children at 49.77%, followed by OBC at 44.40%. Children from the General category fare relatively better, with a stunting rate of 38.83%. These differences likely reflect systemic socioeconomic disadvantages, such as poorer living conditions, lower access to healthcare, and higher food insecurity among marginalized communities.

Religious differences are also notable, with Muslim households reporting a higher stunting rate (46.65%) compared to Hindu households (44.7%). Households in the ‘Others’ category show an exceptionally low stunting rate (5.73%), but this may be due to a very small sample size in this category. These religious differences may be linked to variations in SES, dietary patterns, and health service utilization across religious communities.

A clear urban-rural disparity is seen, with stunting rates in rural areas (46.23%) far exceeding those in urban areas (34.59%). This disparity highlights poorer access to nutrition, healthcare, sanitation, and early childhood development services in rural areas, which contributes to worse growth outcomes for rural children.

Wealth plays a significant protective role against stunting. In the households in the lowest quartile, stunting prevalence is 51.68%, gradually declining to 45.08% in households in the second quartile, 36.07% in middle-income households, 29.94% in households in the fourth quartile, and just 26.92% in households in the highest quartile. This gradient underscores the direct link between economic status and nutritional well-being, with wealthier families better able to afford diverse foods, healthcare, and improved living conditions.

Children of mothers who consumed IFA supplements during pregnancy have a lower stunting rate (40.92%) compared to children whose mothers did not consume IFA (47.01%). This demonstrates that maternal nutrition during pregnancy directly influences child growth, likely through improved birth weight and better overall maternal health. A similar trend is observed with maternal tetanus toxoid (TT) immunization. Children whose mothers received two or more TT shots have a slightly lower stunting rate (41.66%), compared to those whose mothers received fewer than two shots (42.94%). This may reflect broader access to maternal health services, which tend to improve overall maternal and child health.

Finally, stunting rates in the Aspirational Districts (45.08%) are substantially higher than the average for all districts of Uttar Pradesh (39.71%). This finding highlights the disproportionate burden of chronic malnutrition faced by children in these underdeveloped districts, emphasizing the urgent need for focused nutrition, health, and development interventions in these areas.

Overall, child stunting in the Aspirational Districts of Uttar Pradesh is driven by a complex interplay of factors, including district-level development, SES, maternal education, household wealth, birth order, rural residence, and maternal health behaviors. Addressing stunting in these districts will require multi-sectoral interventions that address maternal nutrition, healthcare access, food security, education, and social inequalities.

Logistic Regression Analysis

The logistic regression analysis presented in Table 2 examines the predictors of stunting among children aged 0-59 months in the Aspirational Districts of Uttar Pradesh during 2019-21. Child’s age emerges as a strong predictor of stunting. Compared to children under 12 months of age, children aged 12-23 months are more than twice as likely (OR = 2.25) to be stunted, while those aged 24-59 months are at an even higher risk, being 2.66 times more likely to experience stunting. Both of these findings are highly statistically significant (p<0.001). This clearly indicates that the risk of stunting increases sharply as children grow older, reflecting cumulative nutritional deficiencies as children transition to complementary foods and become increasingly exposed to infections, poor diets, and inadequate health services.

**Table 2:** Predictors of stunting among children aged 0-59 months in aspirational districts of Uttar Pradesh, 2019-21: logistic regression estimates

Predictor Variables	Odds Ratio	Std. Err.	z value	P>|z| (Level of Significance)	[95% Conf. Interval]	
Child's age in months						
<12 (Ref.)	--					
12-23	2.25	0.26	7.13	0.000	1.8	2.82
24-59	2.66	0.27	9.53	0.000	2.17	3.25
Sex of the child						
Male (Ref.)	--					
Female	0.97	0.08	-0.42	0.680	0.83	1.13
Birth Order						
1 (Ref.)	--					
2 or 3	1.14	0.12	1.2	0.230	0.92	1.4
4 or 5	1.33	0.19	2	0.050	1.01	1.77
6 or higher	1.46	0.29	1.9	0.060	0.99	2.16
Mothers age at child birth						
13-24 (Ref.)	--					
25-34	0.86	0.09	-1.54	0.120	0.71	1.04
35-49	0.97	0.17	-0.17	0.870	0.68	1.38
Place of Residence						
Urban (Ref.)	--					
Rural	1.53	0.24	2.7	0.010	1.12	2.09
Mothers Education						
Illiterate (Ref.)	--					
Primary	0.91	0.11	-0.76	0.450	0.71	1.16
Secondary	0.75	0.08	-2.74	0.010	0.62	0.92
Higher	0.58	0.09	-3.52	0.000	0.43	0.79
Religion						
Hindu (Ref.)	--					
Muslim	1.03	0.11	0.24	0.810	0.83	1.26
Other	0.16	0.17	-1.72	0.090	0.02	1.29
Caste						
SC/ST (Ref.)	--					
OBC	0.85	0.08	1.69	0.090	0.71	1.03
Others	0.76	0.1	-2.07	0.040	0.59	0.99
Mother received two or more tetanus injections during pregnancy						
<2 (Ref.)	--					
*≥*2	0.97	0.1	-0.33	0.740	0.8	1.17
Mothers received iron and folic acid tablets during pregnancy						
No (Ref.)	--					
Yes	0.91	0.1	-0.86	0.390	0.74	1.13
Constant	0.33	0.08	-4.43	0.000	0.2	0.54

The analysis reveals no statistically significant difference in stunting risk between male and female children. The OR for female children is 0.97, indicating a slightly lower risk than males, but this result is not significant (p=0.68). This suggests that, at least in these districts, gender does not play a major role in determining stunting risk when other factors are controlled.

Birth order shows a gradual increase in stunting risk with higher birth ranks. Compared to first-born children, children born as the fourth or fifth child have 33% higher odds of being stunted (OR = 1.33, p=0.05). Children born sixth or later have 46% higher odds (OR = 1.46), although this result is only marginally significant (p=0.06). These findings align with established research showing that children in larger families face resource competition, where limited household resources (food, healthcare, attention) are spread thin across more children, increasing vulnerability to undernutrition.

Maternal age at the time of childbirth does not significantly predict stunting in this analysis. Compared to children born to mothers aged 13-24 years, those born to mothers aged 25-34 years show a slightly lower risk (OR = 0.86), but this is not statistically significant (p=0.12). Similarly, children born to mothers aged 35-49 years do not show any clear association with stunting risk (OR = 0.97, p=0.87). These findings suggest that, in this context, maternal age at childbirth does not independently influence stunting after accounting for other factors like education, wealth, and residence.

A child’s place of residence is a significant predictor of stunting. Children living in rural areas are 53% more likely to be stunted (OR = 1.53, p=0.01) compared to their urban counterparts. This confirms that rural areas face significant disadvantages in terms of access to healthcare, nutritional services, dietary diversity, and sanitation infrastructure, all of which contribute to higher rates of chronic malnutrition.

Maternal education shows a strong protective effect against stunting. Compared to children of illiterate mothers, those whose mothers have completed secondary education are at 25% lower risk of stunting (OR = 0.75, p=0.01). The protective effect is even stronger for children of mothers with higher education, who face 42% lower odds of stunting (OR = 0.58, p<0.001). These results highlight the critical role of maternal education in improving child nutrition outcomes, likely through better knowledge of optimal feeding practices, improved health-seeking behavior, and better economic opportunities for more educated mothers.

Religion does not show a statistically significant association with stunting. Compared to Hindu children, Muslim children have a very similar risk of stunting (OR = 1.03, p=0.81). Children from other religions show a much lower odds ratio (OR = 0.16), but this result is only marginally significant (p=0.09), with a wide confidence interval, likely due to a very small sample size for this group. Overall, religious affiliation does not emerge as a clear predictor of stunting in this analysis.

Caste shows some significant differences in stunting risk. Compared to children from SC and ST, children from Other Castes (non-SC/ST/OBC) face 24% lower odds of stunting (OR = 0.76, p=0.04). The OR for OBC children is also lower (OR = 0.85), though this result is only marginally significant (p=0.09). These findings highlight persistent caste-based disparities in child nutrition, with children from historically disadvantaged castes experiencing worse nutritional outcomes.

Whether the mother received two or more TT injections during pregnancy does not show any significant relationship with child stunting (OR = 0.97, p-value = 0.74). This does not mean TT shots are unimportant for maternal and newborn health, but it suggests they do not directly influence long-term child growth. Similarly, maternal consumption of IFA tablets during pregnancy does not show a statistically significant effect on stunting (OR = 0.91, p=0.39). While IFA improves birth outcomes, its direct influence on stunting by five years of age appears limited in this population.

Overall, older children face a significantly higher risk of stunting, with the risk more than doubling after infancy. Higher birth order children (4 or more) face a higher risk of stunting, reflecting resource dilution in larger families. Rural children are significantly more likely to be stunted than urban children. Higher maternal education, particularly secondary and higher education, strongly protects against stunting. Children from higher castes face a lower risk, while SC/ST children are more vulnerable to stunting. Religion, maternal age, and maternal health interventions (IFA use and tetanus shots) do not show statistically significant associations with stunting in this analysis. This analysis underscores the multi-dimensional drivers of stunting in Uttar Pradesh’s Aspirational Districts, with socioeconomic factors (education, caste, residence), demographic factors (age, birth order), and structural disadvantages in rural areas playing key roles. Effective interventions to reduce stunting will need to prioritize maternal education, early childhood nutrition, improved rural health services, and targeted support for vulnerable social groups.

Wasting

Table 1 also presents data on the prevalence of wasting and severe wasting among children aged 0-59 months in the Aspirational Districts of Uttar Pradesh during 2019-21. Wasting refers to low weight-for-height, a clear indicator of acute malnutrition caused by recent inadequate dietary intake, illness, or both. Severe wasting, the most extreme form, places children at a substantially higher risk of mortality and indicates urgent nutritional and medical intervention needs. There are considerable variations in the prevalence of wasting and severe wasting across districts, reflecting differing levels of food security, healthcare access, disease prevalence, and local socio-economic conditions.

Among the districts, Sonbhadra stands out with the highest levels of wasting (26.75%) and severe wasting (17.36%), indicating a severe public health crisis. Other districts with extremely high rates of wasting include Sidharthnagar (24.84%), Balrampur (24.93%), and Chitrakoot (24.82%). These figures suggest that a significant proportion of children in these districts are acutely malnourished. At the lower end, Bahraich records the lowest wasting prevalence (14.35%), but even this figure exceeds global thresholds for public health concern. The data underscores that acute malnutrition is a widespread challenge across all Aspirational Districts.

A clear age-related trend emerges from the data, highlighting how acute malnutrition disproportionately affects the youngest children. Children under the age of 12 months show the highest prevalence of wasting (28.05%) and severe wasting (14.37%), making them the most vulnerable age group. As children grow older, the prevalence of wasting decreases. Among children aged 12-23 months, wasting falls to 23.05% and severe wasting drops to 11.05%. In the 24-59 months age group, wasting declines further to 17.07% and severe wasting to 9.91%. This pattern likely reflects inadequate complementary feeding practices, poor dietary diversity, and exposure to repeated infections in the first two years of life, which are all critical factors contributing to acute malnutrition.

When comparing boys and girls, male children experience slightly higher levels of both wasting (21.31%) and severe wasting (11.28%) compared to females (19.44% and 10.67%, respectively). Although the differences are not dramatic, they could reflect biological factors such as higher metabolic rates in boys or subtle gender biases in feeding practices, where boys may be exposed to more aggressive feeding in response to illness. However, the differences are not large enough to indicate a strong gender disparity.

The relationship between birth order and wasting is less pronounced than for chronic malnutrition (stunting). First-born children have a wasting prevalence of 19.71%, which is slightly lower than children of birth orders two or three (21.51%). However, children with birth orders four to five (18.7%) and those from birth orders six or higher (20.13%) do not show a consistent upward trend. This suggests that, while higher birth order is associated with a higher risk of chronic undernutrition (stunting) due to long-term resource dilution, wasting (a short-term nutritional indicator) is influenced more by recent illnesses, feeding practices, and immediate food security conditions rather than birth order alone.

The mother’s age at childbirth does not show a strong or consistent relationship with wasting. Children born to mothers aged 13-24 years experience a wasting rate of 20.38%. Children of mothers aged 25-34 years show a slightly lower rate (19.8%), while children of mothers aged 35-59 years have the highest wasting rate (23.84%). This pattern suggests that older mothers (age above 35 years) may experience greater economic stress or have larger families, limiting their ability to provide adequate nutrition to younger children. However, maternal age alone is not a dominant predictor of wasting.

Maternal education has a modest protective effect on wasting. Children of illiterate mothers show a wasting prevalence of 21.02%, compared to 16.08% among children whose mothers have higher education. This reduction in risk is likely due to better awareness of appropriate infant feeding practices, improved hygiene knowledge, and better healthcare-seeking behavior among educated mothers. Although the impact of maternal education is not as dramatic for wasting as it is for stunting, education remains an important protective factor.

Differences by caste are present but relatively small for acute malnutrition. Children from SC/ST communities show the highest wasting prevalence at 21.32%, compared to 20.28% among OBC children and 18.87% among children from general caste families. While the differences are not vast, they reflect the structural disadvantages and food insecurity that disproportionately affect marginalized communities, even in acute malnutrition scenarios.

The data reveal no significant differences in wasting prevalence between religious groups. Hindu children have a wasting rate of 20.44%, which is nearly identical to the rate among Muslim children (20.17%). Children in the ‘Other’ religious category report a much lower wasting rate (10.83%), but this is likely due to a very small sample size. Overall, religion is not a major differentiating factor for acute malnutrition.

Interestingly, wasting is more prevalent in urban areas (26.88%) than in rural areas (19.63%). This is a somewhat unexpected finding, as rural areas typically experience greater food insecurity. This anomaly could reflect greater awareness and treatment-seeking in urban areas, leading to better detection of wasting.

There is no clear linear relationship between wealth and wasting. The children from households in the lowest wealth quartile experience a wasting rate of 21.29%, higher than that of some wealthier groups. However, children in the households in the highest wealth quartile also have a substantial wasting rate of 18.28%, indicating that acute malnutrition is not confined to poorer families. This suggests that immediate factors like feeding practices, child illnesses, and healthcare access play a bigger role in wasting than wealth alone.

Children whose mothers received IFA tablets during pregnancy paradoxically show higher wasting rates (22.31%) compared to children whose mothers did not (19.31%). This could reflect selection bias, as mothers at higher nutritional risk may have been targeted for supplementation. Similarly, maternal TT immunization shows no significant impact on wasting, with nearly identical rates for children of mothers who received less than two TT injections (21.88%) and those who received two or more (21.79%). These findings suggest that while antenatal interventions improve maternal and newborn health, they are not directly protective against acute malnutrition in the first five years.

Children in the Aspirational Districts of Uttar Pradesh experience higher wasting rates (20.35%) compared to the state average of 17.32%. This gap highlights the particular vulnerability of children in these underserved districts, where poverty, weak health infrastructure, and food insecurity combine to create a heightened risk of acute malnutrition.

Logistic Regression Analysis

The logistic regression analysis presented in Table 3 identifies the key predictors associated with wasting, a form of acute malnutrition characterized by low weight-for-height among children aged 0-59 months in the Aspirational Districts of Uttar Pradesh. Wasting reflects recent nutritional deprivation or illness and places children at an elevated risk of mortality.

**Table 3:** Predictors of wasting among children aged 0-59 months in aspirational districts of Uttar Pradesh, 2019-21: logistic regression estimates

Predictor Variables	Odds Ratio	Std. Err.	z	P>|z| (Level of Significance)	[95% Conf. Interval]	
Childs age in months						
<12 (Ref.)	--					
12-23	0.76	0.09	-2.22	0.030	0.6	0.97
24-59	0.53	0.06	-5.64	0.000	0.42	0.66
Sex of the child						
Male (Ref.)	--					
Female	0.94	0.09	-0.66	0.510	0.78	1.13
Birth Order						
1 (Ref.)	--					
2 or 3	1.05	0.13	0.39	0.700	0.82	1.34
4 or 5	0.87	0.15	-0.79	0.430	0.63	1.22
6 or higher	0.81	0.2	-0.86	0.390	0.51	1.3
Mothers age at child birth						
13-24 (Ref.)	--					
25-34	1.12	0.13	0.94	0.350	0.89	1.4
35-49	1.13	0.24	0.57	0.570	0.74	1.72
Place of Residence						
Urban (Ref.)	--					
Rural	0.63	0.11	-2.76	0.010	0.46	0.88
Mothers Education						
Illiterate (Ref.)	--					
Primary	1.00	0.14	-0.02	0.990	0.75	1.33
Secondary	0.79	0.1	-1.97	0.050	0.62	1
Higher	0.57	0.11	-2.93	0.000	0.4	0.83
Religion						
Hindu (Ref.)	--					
Muslim	0.99	0.12	-0.11	0.910	0.77	1.26
Other	0.96	0.78	-0.05	0.960	0.2	4.72
Caste						
SC/ST (Ref.)	--					
OBC	0.82	0.09	-1.86	0.060	0.66	1.01
Others	0.87	0.13	-0.92	0.360	0.64	1.17
Mother received two or more tetanus injections during pregnancy						
<2 (Ref.)	--					
*≥*2	0.96	0.11	-0.35	0.720	0.77	1.2
Mothers received iron and folic acid tablets during pregnancy						
No (Ref.)	--					
Yes	1.09	0.14	0.64	0.530	0.84	1.4
Constant	0.77	0.21	-0.93	0.350	0.45	1.33

Child’s age is a significant and strong predictor of wasting. Compared to infants under the age of 12 months, children aged 12-23 months have 24% lower odds of wasting (OR = 0.76), with this result being statistically significant (p=0.03). The risk continues to decline with age, with children aged 24-59 months showing 47% lower odds of wasting (OR = 0.53, p<0.001). This trend highlights the particular vulnerability of infants under 12 months to acute malnutrition, which is likely due to suboptimal breastfeeding and the introduction of inappropriate or insufficient complementary foods after the first six months of life. The early months represent a critical window for targeted nutritional interventions, including promotion of exclusive breastfeeding and proper complementary feeding practices.

The analysis finds no statistically significant difference in wasting risk between male and female children. Compared to male children, female children have a slightly lower odds of wasting (OR = 0.94), but this effect is not statistically significant (p=0.51). This indicates that gender alone does not play a major role in determining acute malnutrition in the Aspirational Districts once other factors are taken into account.

Birth order does not show a strong or consistent association with wasting. Compared to first-born children, those with birth orders of 2 or 3 have nearly the same odds of being wasted (OR = 1.05, p=0.70). Children from higher birth orders, such as the fourth or fifth child (OR = 0.87) and sixth or later child (OR = 0.81), show somewhat lower odds of wasting, but these differences are not statistically significant. This finding suggests that, while higher birth order is associated with increased risk of chronic undernutrition (stunting) due to long-term resource dilution, it is less relevant for acute malnutrition, which is more influenced by immediate dietary intake and recent illness.

The mother’s age at childbirth does not emerge as a significant predictor of wasting. Compared to children born to mothers aged 13-24 years, children born to mothers aged 25-34 years show slightly higher odds of wasting (OR = 1.12), but this result is not statistically significant (p=0.35). Similarly, children born to mothers aged 35-49 years show 13% higher odds of wasting (OR = 1.13), but this relationship is also not statistically significant (p=0.57). These findings suggest that maternal age at childbirth, in isolation, is not a major driver of acute malnutrition in these districts.

Place of residence emerges as a significant and surprising predictor of wasting. Compared to children living in urban areas, children in rural areas have 37% lower odds of being wasted (OR = 0.63, p=0.01). This unexpected finding challenges the conventional assumption that rural children are more vulnerable to malnutrition. It may reflect the rising levels of food insecurity, poorer sanitation, and greater disease burden in low-income urban settlements, where dietary diversity and feeding practices are often suboptimal. This finding highlights the need for greater attention to the nutrition challenges faced by urban children, particularly those living in slums and informal settlements.

Maternal education shows a strong protective effect against wasting, reinforcing the importance of educating mothers to improve child health outcomes. Compared to children of illiterate mothers, children of mothers with secondary education have 21% lower odds of wasting (OR = 0.79, p=0.05), a result that is statistically significant. The protective effect is even greater for children of mothers with higher education, who face 43% lower odds of wasting (OR = 0.57, p=0.00). This pattern underscores how maternal education improves feeding practices, hygiene awareness, healthcare utilization, and food security within the household, all of which reduce the risk of acute malnutrition.

Religious affiliation does not show any statistically significant relationship with wasting. Compared to Hindu children, Muslim children have nearly identical odds of being wasted (OR = 0.99, p=0.91). Similarly, children from ‘Other’ religious groups show no significant difference (OR = 0.96, p=0.96), though the wide confidence interval for this group reflects a small sample size. This confirms that religion is not a strong determinant of acute malnutrition once other factors are considered.

Caste shows only a marginal relationship with wasting risk. Compared to children from SC and ST households, children from OBC households have 18% lower odds of wasting (OR = 0.82), a result that is just outside conventional statistical significance (p=0.06). Children from ‘Other’ caste groups show 13% lower odds of wasting (OR = 0.87), but this is not statistically significant (p=0.36). This suggests that wasting, being a more immediate indicator of nutritional stress, may cut across caste lines more evenly than stunting, although marginalized groups like SC/ST children still face slightly elevated risk.

Receiving two or more TT injections during pregnancy does not show a statistically significant effect on wasting (OR = 0.96, p=0.72). Children whose mothers received IFA supplements during pregnancy show slightly higher odds of wasting (OR = 1.09), but this effect is not statistically significant (p=0.53). These results indicate that, while maternal health interventions are essential for maternal and newborn health, they do not directly protect against acute malnutrition in the later months and years of life.

Overall, the risk of wasting among children aged 0-59 months in the Aspirational Districts of Uttar Pradesh is most strongly influenced by, the child’s age, with the youngest children (under 12 months) being at highest risk, place of residence, where urban children face significantly higher risk than rural children, maternal education, which offers a strong protective effect, particularly when mothers have secondary or higher education. In contrast, factors such as birth order, maternal age at birth, caste, religion, and maternal health interventions (IFA and TT injections) do not show strong or consistent associations with acute malnutrition. These findings underscore the importance of targeting infant feeding practices, urban nutrition challenges, and maternal education programs to effectively combat wasting in the most vulnerable populations.

Underweight

Table 1 also highlights the prevalence of underweight and severely underweight children aged 0-59 months in the Aspirational Districts of Uttar Pradesh during 2019-21. Being underweight, which refers to low weight-for-age, is a composite indicator reflecting both chronic undernutrition (stunting) and acute undernutrition (wasting). Children classified as severely underweight face extremely high risks of illness, developmental delays, and mortality, making this an important public health concern.

The prevalence of underweight and severe underweight varies significantly across districts within the Aspirational Districts of Uttar Pradesh. The highest prevalence of underweight children is reported in Sonbhadra, where 46.49% of children are underweight, followed closely by Chitrakoot at 41.78% and Shrawasti at 40.76%. These districts also show alarmingly high rates of severely underweight children, with Chitrakoot reporting 19.61% and Balrampur reporting 19.96%. On the lower end of the spectrum, Chandauli has the lowest underweight prevalence at 29.94%, although this still exceeds acceptable levels and remains a cause for concern. These inter-district differences reflect the diverse socioeconomic conditions, food security levels, healthcare access, and disease burden, indicating the need for district-specific strategies to address malnutrition.

Age-related patterns in the data reveal that the proportion of underweight children increases with age. Among children under 12 months, 28.11% are underweight, rising to 35.66% for those aged 12-23 months, and further to 42.32% for children aged 24-59 months. Severe underweight follows the same trajectory, increasing from 13.53% among infants under 12 months to 16.74% among children aged 24-59 months. This age gradient underscores the increasing vulnerability of children as they grow older, particularly as they transition from exclusive breastfeeding to complementary foods and become more exposed to infections, inadequate diets, and insufficient healthcare.

The data also reflects slight differences in nutritional outcomes by sex. Male children show a higher prevalence of underweight (38.95%) compared to female (37.12%), with a similar pattern for severe underweight, where 16.79% of boys are severely underweight compared to 14.43% of girls. These differences are relatively small, indicating no pronounced gender bias in nutritional status, although boys may face slightly greater biological vulnerability due to higher energy needs or differences in care-seeking behavior during illness episodes.

Birth order also emerges as a contributing factor to underweight prevalence. First-born children show a lower prevalence of underweight (36.64%) compared to those born fourth or fifth (41.12%) and those with a birth order of six or more (40%). This increasing trend is consistent with the resource dilution hypothesis, where larger family sizes limit the availability of household resources, including food, parental attention, and healthcare, thereby increasing the risk of malnutrition for children born later.

Maternal age at childbirth does not show a very clear or consistent relationship with underweight prevalence, though some patterns emerge. Children born to younger mothers aged 13-24 years have the highest underweight prevalence (38.84%), followed closely by children born to older mothers aged 35-59 years (40.53%). Children born to mothers aged 25-34 years show a slightly lower underweight rate at 36.97%. These patterns suggest that both younger mothers, who may lack knowledge and experience, and older mothers, who may face higher parity and economic pressures, contribute to higher underweight risk among their children.

Maternal education emerges as one of the most important protective factors against underweight. Children born to illiterate mothers experience the highest underweight prevalence at 42.92%, while those whose mothers have attained higher education face a dramatically lower underweight prevalence of 26.34%. A similar protective trend is seen for severe underweight, declining from 18.29% among children of illiterate mothers to 11.28% among children whose mothers have received higher education. This relationship reflects the critical role of maternal education in enhancing awareness of optimal feeding practices, improving hygiene and healthcare-seeking behavior, and boosting household economic opportunities, all of which help protect children from undernutrition.

Social identity also plays an important role, with children belonging to SC/ST households experiencing the highest underweight prevalence at 43.4%, followed by OBC at 37.17%. In contrast, children from General category households fare better, with an underweight prevalence of 31.59%. The same pattern is seen for severely underweight children, where SC/ST children experience the highest rates. These findings highlight the persistent impact of social exclusion, economic marginalization, and unequal access to services on nutritional outcomes among historically disadvantaged communities.

When analyzed by religion, the data show minimal differences between Hindu and Muslim households, with underweight prevalence at 38.18% for Hindu children and 37.93% for Muslim children. The “Others” category, which is likely a very small sample, reports a much lower underweight rate of 16.56%, although this figure should be interpreted with caution due to sample size limitations. Overall, religious identity does not emerge as a significant determinant of underweight risk, unlike socio-economic status, caste, and maternal education.

There is a clear rural-urban divide in underweight prevalence. In rural areas, 38.71% of children are underweight, compared to 32.14% in urban areas. This difference reflects poorer access to nutrition, healthcare, and sanitation services in rural areas, along with higher levels of poverty and food insecurity. The rural-urban gap is slightly smaller for severe underweight, with 15.69% in rural areas compared to 15.25% in urban areas.

Household wealth is strongly and consistently linked to underweight prevalence. Among children from the households in the lowest wealth quartile, 43.65% are underweight, compared to only 18.5% among children from the households in the highest quartile. Severe underweight follows the same pattern, declining from 17.82% among the households in the lowest quartile to 8.38% among the households in the highest quartile. This reinforces the direct connection between household economic capacity and child nutritional status, where wealthier families are better able to provide diverse, nutritious foods, access to healthcare, and create healthier home environments.

Maternal health interventions, such as IFA supplementation during pregnancy, are associated with slightly lower underweight rates. Children of mothers who received IFA have an underweight prevalence of 35.4%, compared to 38.01% for those whose mothers did not receive supplements. A similar, though modest, effect is observed for severe underweight. This indicates that maternal nutrition during pregnancy contributes modestly to better child nutritional outcomes. The number of TT injections received during pregnancy does not show any meaningful association with underweight prevalence, with nearly identical rates for children whose mothers received fewer than two injections and those whose mothers received two or more.

Across all Aspirational Districts, 38.07% of children are underweight, compared to 32.14% for Uttar Pradesh overall. Similarly, 15.65% of children in Aspirational Districts are severely underweight, compared to 11.13% across the state. These figures underscore the urgent need for targeted nutritional interventions in these underdeveloped districts, where the burden of undernutrition is both higher and more severe.

Logistic Regression Analysis

The logistic regression analysis presented in Table 4 examines the factors influencing the likelihood of children aged 0-59 months being underweight in the Aspirational Districts of Uttar Pradesh.

**Table 4:** Predictors of underweight among children aged 0-59 months in aspirational districts of Uttar Pradesh, 2019-21: logistic regression estimates

Predictor Variables	Odds Ratio	Std. Err.	z	P>|z| (Level of Significance)	[95% Conf. Interval]	
Child's age in months						
<12 (Ref.)	--					
12-23	1.35	0.15	2.72	0.010	1.09	1.68
24-59	1.59	0.16	4.72	0.000	1.31	1.92
Sex of the child						
Male (Ref.)	--					
Female	0.94	0.08	-0.76	0.450	0.81	1.1
Birth Order						
1 (Ref.)	--					
2 or 3	0.93	0.1	-0.7	0.480	0.75	1.14
4 or 5	1.01	0.14	0.05	0.960	0.76	1.33
6 or higher	0.98	0.19	-0.11	0.910	0.66	1.44
Mothers age at child birth						
13-24 (Ref.)	--					
25-34	0.91	0.09	-0.93	0.350	0.75	1.11
35-49	1.05	0.18	0.28	0.780	0.75	1.48
Place of Residence						
Urban (Ref.)	--					
Rural	1.16	0.18	0.94	0.350	0.85	1.57
Mothers Education						
Illiterate (Ref.)	--					
Primary	0.87	0.11	-1.08	0.280	0.69	1.11
Secondary	0.77	0.08	-2.5	0.010	0.63	0.95
Higher	0.56	0.09	-3.66	0.000	0.42	0.77
Religion						
Hindu (Ref.)	--					
Muslim	0.99	0.1	-0.05	0.960	0.81	1.22
Other	0.91	0.66	-0.13	0.900	0.22	3.75
Caste						
SC/ST (Ref.)	--					
OBC	0.89	0.08	-1.24	0.220	0.75	1.07
Others	0.69	0.09	-2.81	0.010	0.53	0.89
Mother received two or more tetanus injections during pregnancy						
<2 (Ref.)	--					
* ≥*2	0.99	0.1	-0.08	0.930	0.82	1.2
Mothers received iron and folic acid tablets during pregnancy						
No (Ref.)	--					
Yes	0.92	0.1	-0.76	0.450	0.75	1.14
Constant	0.57	0.14	-2.3	0.020	0.36	0.92

One of the most notable findings is the strong association between a child’s age and underweight status. Compared to children under 12 months of age, children aged 12-23 months are 1.35 times more likely to be underweight, with this relationship being statistically significant (p=0.01). The risk of underweight continues to increase for older children, with those aged 24-59 months being 1.59 times more likely to be underweight compared to infants, a highly significant finding (p<0.001). This confirms that the risk of underweight accumulates with age, reflecting the progressive nutritional vulnerability children experience as they grow older, particularly in environments with inadequate complementary feeding, recurrent infections, and poor access to healthcare.

The sex of the child does not emerge as a statistically significant predictor of underweight. Female children are slightly less likely to be underweight compared to males, with an odds ratio of 0.94, but this difference is not statistically significant (p=0.45). This suggests that there is no strong gender bias in underweight risk in these districts once other factors are accounted for.

Birth order also shows no statistically significant association with underweight risk. Compared to first-born children, those with birth orders of 2 or 3 have a slightly lower odds ratio of 0.93, while those born fourth or fifth have an odds ratio of 1.01, essentially no difference. Similarly, children with a birth order of six or higher have an odds ratio of 0.98. These findings indicate that birth order alone does not independently predict underweight status in these districts, despite its recognized role in chronic malnutrition in other contexts.

The mother’s age at childbirth is also not significantly associated with the risk of underweight. Compared to children born to mothers aged 13-24 years, those born to mothers aged 25-34 years have a slightly lower OR of 0.91, while those born to mothers aged 35-49 years have a slightly higher OR of 1.05. Both relationships are statistically insignificant, suggesting that maternal age at birth does not play a major role in determining underweight risk once other factors are controlled.

Place of residence, whether the child lives in a rural or urban area, does not show a statistically significant association with underweight risk either. Children living in rural areas have 1.16 times higher odds of being underweight compared to urban children, but this result is not statistically significant (p=0.35). This indicates that the rural-urban divide in underweight prevalence, seen in descriptive statistics, may be explained by other socio-economic and demographic factors rather than residence alone.

Maternal education is one of the strongest protective factors against underweight, with a clear gradient of decreasing risk as maternal education increases. Compared to children of illiterate mothers, children whose mothers have secondary education have 23% lower odds of being underweight (OR = 0.77, p=0.01), while those whose mothers have higher education have 44% lower odds (OR = 0.56, p<0.001). This confirms that better-educated mothers are more likely to have improved knowledge of appropriate feeding practices, better healthcare-seeking behavior, and greater economic opportunities, all of which contribute to better child nutrition.

Religious affiliation does not have any significant impact on underweight risk. Compared to Hindu children, Muslim children have almost identical odds (OR = 0.99), with no statistical significance (p=0.96). Children belonging to other religions also show no significant difference (OR = 0.91, p=0.90), though the wide confidence interval for this group suggests a very small sample size, making the estimate less reliable. Overall, this confirms that religion is not a major predictor of underweight risk once other factors are considered.

Caste, on the other hand, does show a significant association with underweight risk. Compared to children from SC/ST households, children from OBC households have 11% lower odds of being underweight (OR = 0.89), though this result is not statistically significant (p=0.22). However, children from the General category experience 31% lower odds of being underweight (OR = 0.69, p=0.01). This significant reduction highlights the ongoing disadvantage faced by children from historically marginalized caste groups, where poverty, social exclusion, and reduced access to health and nutrition services contribute to higher underweight prevalence.

The model finds no significant association between maternal TT injection coverage and underweight risk. Children whose mothers received two or more TT injections during pregnancy have nearly identical odds of being underweight (OR = 0.99) compared to those whose mothers received fewer than two injections, with no statistical significance (p=0.93). This confirms that tetanus immunization, while important for maternal and neonatal health, does not directly influence longer-term nutritional outcomes like underweight. Similarly, maternal consumption of IFA supplements during pregnancy is not significantly associated with underweight risk. Children of mothers who received IFA have 8% lower odds of being underweight (OR = 0.92), but this relationship is not statistically significant (p=0.45). This suggests that while maternal nutrition is critical for good birth outcomes, its direct influence on underweight prevalence diminishes as children age and their nutrition depends more on feeding practices and overall household conditions.

This analysis highlights that a child’s age, maternal education, and caste are the most important predictors of underweight status in the Aspirational Districts of Uttar Pradesh. The risk of underweight rises sharply with age, indicating cumulative nutritional deficits as children grow older. Higher maternal education provides strong protection, emphasizing the importance of improving female education as a long-term strategy to reduce child undernutrition. Additionally, children from socially disadvantaged castes remain at significantly higher risk, reflecting the need for targeted social protection, improved health services, and economic opportunities for marginalized communities. In contrast, factors such as sex, birth order, maternal age, religion, and rural-urban residence do not show statistically significant effects, indicating that underweight risk in these districts is shaped more by socioeconomic inequalities and maternal education than by biological or geographic factors.

Overweight

Table 1 also presents data on the prevalence of overweight among children aged 0-59 months in Aspirational Districts of Uttar Pradesh (2019-21). The indicators used, overweight for height and overweight for age, capture excess weight relative to a child’s height or age, signalling imbalanced nutrition, poor dietary patterns, or other lifestyle factors. Though overweight remains far less prevalent than underweight, the data reveal some emerging patterns that deserve attention, particularly as Uttar Pradesh’s nutrition landscape evolves.

Overweight prevalence varies considerably across districts. In terms of overweight for height, the highest prevalence is found in Chitrakoot, where 6.72% of children are overweight for their height. This is followed by Bahraich at 4.13% and Balrampur at 3.9%. In contrast, districts such as Sonbhadra (0.87%) and Chandauli (0.57%) report extremely low levels of overweight for height.

When looking at overweight for age, levels are significantly lower across all districts, indicating that fewer children have excess weight relative to their age. The highest prevalence of overweight for age is seen in Balrampur (1.92%) and Chitrakoot (1.54%), with several districts such as Chandauli, Fatehpur, Shrawasti, Sidharthnagar, and Sonbhadra reporting levels below 1%. In some cases, especially in districts like Chandauli, the numbers are so low they are marked with an asterisk, indicating extremely small sample sizes that should be interpreted with caution. These figures confirm that, while overweight is present, it remains a relatively minor concern compared to undernutrition in these districts.

The prevalence of overweight decreases with age, demonstrating a clear age-related pattern. Among children under 12 months, 5.98% are overweight for height, but this falls to 3.82% among those aged 12-23 months, and further to 2.11% among children aged 24-59 months. This pattern likely reflects differences in feeding practices during infancy, where overfeeding and inappropriate complementary feeding may lead to early excess weight gain, which tapers off as children grow older and become more mobile. The same pattern holds for overweight for age, which starts at 2.46% for infants under 12 months and declines sharply to 0.43% for children aged 24-59 months. This highlights that early life feeding practices, especially formula use, overfeeding, and sugary or energy-dense complementary foods, could be contributing factors to early overweight.

The data reveal no significant difference between male and female children in terms of overweight prevalence. Among males, 3.12% are overweight for height compared to 3.24% of female children. Similarly, 0.93% of male children are overweight for age compared to 0.96% of female children. This indicates that, unlike undernutrition, which often shows slight gender biases, overweight does not appear to be gendered in this population.

There is some variability in overweight prevalence by birth order, but the pattern is not linear. Among first-born children, 3.15% are overweight for height, with this figure dipping slightly for children of birth order two or three (2.6%). Interestingly, children of birth order four to five have the highest overweight for height prevalence at 4.58%, while those born sixth or later show a lower prevalence of 3.4%. These fluctuations may reflect differences in family feeding practices, access to resources, and changing parental attitudes over successive births. Overweight for age does not follow the same pattern, with the highest rates seen among first-borns (1.11%) and lower rates for higher birth orders, but the differences are minor.

There is some indication that children born to older mothers are more likely to be overweight for height. Children born to mothers aged 13-24 years have an overweight for height prevalence of 2.68%, which rises to 3.39% for children of mothers aged 25-34 years, and peaks at 5.27% for children of mothers aged 35-59 years. This could reflect differences in feeding practices among older mothers, who may be more likely to overfeed or introduce energy-dense foods earlier. Interestingly, for overweight for age, there is no clear trend, with children of younger and older mothers showing similar rates around 0.85-1.06%.

Surprisingly, overweight for height is most common among children of mothers with higher education. Among children of mothers with higher education, 5.16% are overweight for height, compared to just 2.33% for children of mothers with primary education and 2.36% for children of mothers with secondary education. This suggests that better-educated mothers, despite their advantages in avoiding undernutrition, may inadvertently contribute to overnutrition through overfeeding, reliance on packaged foods, or misperceptions of ideal body weight. Overweight for age follows a similar pattern, with the highest rates (1.35%) among children of mothers with higher education, and the lowest (0.35%) among children of mothers with primary education.

Overweight for height also varies by social category, with the highest prevalence among General category children (4.02%) compared to SC/ST children (2.68%) and OBC children (3.18%). This pattern suggests that higher SES and better household food security may contribute to higher overweight risk in these groups. A similar trend is seen for overweight for age, which is also highest among the General category children (1.48%).

Religious differences also emerge in overweight prevalence. Among Hindu children, 2.81% are overweight for height, compared to 4.13% of Muslim children. This religious gap is mirrored in overweight for age, where 1.16% of Muslim children are overweight, compared to 0.86% of Hindu children. Notably, children classified under ‘Other’ religions show an exceptionally high overweight for height rate of 18.7%, though this is likely due to a very small sample size, making the result unreliable.

Rural children are more likely to be overweight than urban children. In rural areas, 3.37% of children are overweight for height, compared to just 1.26% in urban areas. The same pattern holds for overweight for age, where 0.98% of rural children are overweight compared to 0.52% of urban children. This finding may reflect changing dietary patterns in rural areas, including increased availability of processed foods, coupled with lower physical activity levels.

The relationship between household wealth and overweight is not entirely linear. Among children from the households in the lowest wealth quartile, 3.39% are overweight for height, compared to just 2.48% among the households in the second wealth quartile. Interestingly, the households in the highest quartile see a slight increase to 2.68%, possibly indicating a dual burden of malnutrition in wealthier families, where both undernutrition and overnutrition coexist. Overweight for age follows a similar inconsistent pattern, though the households in the highest quartile show the highest rate (1.4%).

Maternal health interventions such as IFA supplementation and tetanus injections show no clear protective effect against overweight. Children whose mothers received IFA tablets during pregnancy actually show higher overweight for height (4.01%) than children of mothers who did not (3.68%). Similarly, children of mothers who received two or more TT injections are less likely to be overweight for height (3.47%) compared to those who received fewer than two shots (5.7%). This may reflect broader health and socioeconomic differences among mothers who receive full antenatal care.

Overall, overweight for height in the Aspirational Districts is 3.2%, slightly higher than the state average of 3.1%. Overweight for age in the Aspirational Districts stands at 0.93%, compared to 0.79% for all districts in Uttar Pradesh.

## Discussion

The high prevalence of stunting, wasting, and underweight among children aged 0-59 months in the Aspirational Districts of Uttar Pradesh underscores the persistent burden of malnutrition in the state’s most vulnerable regions. These findings are consistent with broader national and international evidence, which has documented the deep interlinkages between poverty, food insecurity, poor maternal health, inadequate child feeding practices, and the high burden of infectious diseases in perpetuating malnutrition in low-resource settings. 

The high prevalence of stunting, particularly among older children, reflects cumulative nutritional deprivation and chronic exposure to poor diets and repeated infections [5].

Previous research has shown that maternal education is a strong protective factor against stunting [6]. Consistent with this, children of mothers with secondary or higher education in the Aspirational Districts had significantly lower stunting rates compared to those born to illiterate mothers. Maternal education is closely linked with better knowledge of optimal infant feeding practices, improved hygiene, and more frequent healthcare-seeking behavior [12]. Moreover, caste-based disparities in stunting observed in the data reflect structural inequalities that have been well-documented in Indian studies [7], with children from SC and ST households experiencing higher rates of stunting due to historically limited access to economic resources, health services, and adequate nutrition.

The increasing prevalence of stunting with age, rising sharply between 12 and 23 months, mirrors findings from global literature, including studies in South Asia, which emphasize that the critical window for preventing stunting is the first 1,000 days from conception to a child’s second birthday. Failure to ensure adequate nutrition during this period results in long-term growth faltering, cognitive deficits, and increased risk of chronic diseases in adulthood [4].

The findings on wasting, the most immediate and visible form of undernutrition, point to high levels of acute malnutrition across the Aspirational Districts. The prevalence of wasting in the Aspirational Districts (20.35%) far exceeds the 15% threshold defined by WHO as a critical public health emergency [10]. This is in line with findings from NFHS-5, which showed that 19.5% of children under five years of age were wasted in Uttar Pradesh [2], indicating that acute malnutrition remains a pressing and unresolved issue in the state.

Wasting prevalence was found to be highest among infants under 12 months, declining gradually with age. This early-life peak in wasting is consistent with previous research demonstrating that poor breastfeeding practices, early introduction of inappropriate complementary foods, and high rates of infections during the first year of life contribute to rapid weight loss [11]. Studies have consistently linked poor dietary diversity, suboptimal breastfeeding practices, and recurrent diarrhea and respiratory infections to increased risk of wasting in Indian infants [9,19].

The counterintuitive finding that urban children in the Aspirational Districts face higher rates of wasting than rural children is also reflected in some recent studies that highlight the emergence of urban malnutrition due to poor dietary patterns, rising food insecurity in urban slums, and limited access to health services for vulnerable urban populations [6]. This evidence underscores the need to expand nutrition and health interventions to urban poor settlements, which are often neglected in rural-focused programs.

The high rates of underweight, particularly among older children, those from poorer households, and children born to illiterate mothers, closely reflect findings from past surveys, including NFHS-5, which showed that 32.1% of children under five years of age were underweight in Uttar Pradesh [2]. In the Aspirational Districts, however, the underweight burden was even higher, at 38.07%, confirming that these economically and socially marginalized districts bear a disproportionate burden of malnutrition.

As with stunting and wasting, the protective effect of maternal education on underweight rates has been documented in numerous Indian and global studies [8]. Mothers with higher education levels are better equipped to adopt appropriate feeding and care practices, engage with health systems, and secure better diets for their children [13].

The clear wealth gradient observed in underweight prevalence in the Aspirational Districts, with children from the households in the lowest wealth quartiles being twice as likely to be underweight compared to those in the highest quartile, echoes long-standing findings that poverty is a root cause of child malnutrition. Poverty limits access to nutritious foods, safe water, sanitation, and healthcare, all of which are crucial for preventing undernutrition [1].

Although the primary nutritional challenge in the Aspirational Districts remains undernutrition, the data on overweight for height and age point to the early stages of a double burden of malnutrition. This is consistent with national and global patterns showing that overweight and obesity are increasingly affecting children in low-income settings, often coexisting with undernutrition within the same communities and even within the same households [15]. This shift is driven by changing dietary habits, increased consumption of processed foods, and reduced physical activity [16]. The higher prevalence of overweight among children of more educated mothers aligns with studies showing that higher-income and more educated families often transition to energy-dense diets more quickly, even while maintaining traditional feeding practices for younger children [14].

A limitation of the study is that the analysis was based on a cross-sectional dataset. In order to assess the causal relationship, a longitudinal study would have been better. 

## Conclusions

This analysis of stunting, wasting, underweight, and overweight among children aged 0-59 months in the Aspirational Districts of Uttar Pradesh highlights the persistent and multifaceted burden of child malnutrition in these disadvantaged regions, driven by socioeconomic, demographic, and health system factors. 

The persistently high prevalence of stunting points to long-term nutritional deprivation and repeated exposure to infections during the critical early years of life. It confirms that chronic malnutrition is deeply entrenched, particularly in rural areas, poorer households, and marginalized caste groups. Children born to uneducated mothers and those from SC/ST households bear the highest burden, highlighting a strong influence of socioeconomic disadvantage and systemic inequalities on child growth outcomes, reinforcing the importance of empowering mothers through education, awareness, and economic opportunities. The extremely high levels of wasting observed reinforce that acute malnutrition remains a severe public health emergency in the Aspirational Districts. The unexpectedly higher prevalence of wasting in urban areas highlights the emerging vulnerability of children living in urban slums. Underweight, which combines elements of both stunting and wasting, remains the most widespread form of malnutrition in the Aspirational Districts. The cumulative impact of long-term food insecurity, poor dietary diversity, repeated infections, and limited access to healthcare contributes to the high rates of underweight across all districts. Although overweight remains relatively uncommon, its emergence signals the beginning of a nutrition transition. As dietary patterns shift and energy-dense processed foods become more accessible, the coexistence of underweight and overweight within the same communities, and even within the same households, is likely to increase. This dual burden of malnutrition presents a new policy challenge, requiring education on healthy feeding practices that address both undernutrition and overnutrition, particularly in urban areas and higher-income families.

In summary, the Aspirational Districts of Uttar Pradesh represent both the greatest challenge and the greatest opportunity for improving child nutrition outcomes in India. By addressing the multiple and overlapping drivers of stunting, wasting, underweight, and overweight, Uttar Pradesh can make significant strides towards achieving the Sustainable Development Goals related to ending hunger, improving health, and reducing inequalities. Improving infant and young child feeding practices, ensuring complete immunization, and promoting hygiene and sanitation are essential for addressing the root causes of chronic malnutrition in these districts. 
